# Neurological complications associated with emerging viruses in Brazil

**DOI:** 10.1002/ijgo.13050

**Published:** 2020-01-23

**Authors:** Jussara R. Angelo, Trevon L. Fuller, Bianca B.S. Leandro, Heitor L.F. Praça, Renata D. Marques, João M.C. Ferreira, Camila C.B. Pupe, Olívia C. Perez, Karin Nielsen‐Saines, Osvaldo J.M. Nascimento, Paulo C. Sabroza

**Affiliations:** ^1^ Samuel Pessoa Department of Endemic Disease Sergio Arouca National School of Public Health Oswaldo Cruz Foundation Rio de Janeiro Brazil; ^2^ Institute of the Environment and Sustainability University of California Los Angeles Los Angeles CA USA; ^3^ Joaquim Venancio National Health Polytechnic School Oswaldo Cruz Foundation Rio de Janeiro Brazil; ^4^ Department of Neurology Federal Fluminense University Niterói, Rio de Janeiro Brazil; ^5^ Department of Political Science Federal University of Piauí Teresina Brazil; ^6^ Pediatric Infectious Diseases David Geffen UCLA School of Medicine Los Angeles CA USA

**Keywords:** Arboviruses, Guillain‐Barré syndrome, Human influenza, Peripheral nervous system diseases, Polyneuropathies, Public hospitals, Sentinel surveillance

## Abstract

**Objective:**

To test the hypotheses that emerging viruses are associated with neurological hospitalizations and that statistical models can be used to predict neurological sequelae from viral infections.

**Methods:**

An ecological study was carried out to observe time trends in the number of hospitalizations with inflammatory polyneuropathy and Guillain‐Barré syndrome (GBS) in the state of Rio de Janeiro from 1997 to 2017. Increases in GBS from month to month were assessed using a Farrington test. In addition, a cross‐sectional study was conducted analyzing 50 adults hospitalized for inflammatory polyneuropathies from 2015 to 2017. The extent to which Zika virus symptoms explained GBS hospitalizations was evaluated using a calibration test.

**Results:**

There were significant increases (Farrington test, *P*<0.001) in the incidence of GBS following the introduction of influenza A/H1N1 in 2009, dengue virus type 4 in 2013, and Zika virus in 2015. Of 50 patients hospitalized, 14 (28.0%) were diagnosed with arboviruses, 9 (18.0%) with other viruses, and the remainder with other causes of such neuropathies. Statistical models based on cases of emerging viruses accurately predicted neurological sequelae, such as GBS.

**Conclusion:**

The introduction of novel viruses increases the incidence of inflammatory neuropathies.

## Abstract 2

The introduction of novel viruses increases the incidence of inflammatory neuropathies in Brazil.

## 1 INTRODUCTION

Guillain‐Barre syndrome (GBS) is a neuromotor disorder typically preceded by infection. GBS is characterized by ascendant, bilateral muscle weakness with depressed tendon reflexes.^1^ Approximately 25% of GBS cases result in respiratory distress requiring mechanical ventilation.^2^ Treatment involves plasma exchange or intravenous immunoglobulin. Although the most frequent causes of GBS are *Campylobacter jejuni*,* Cytomegalovirus*, and influenza A, the disorder has also been associated with dengue virus and Zika virus.^3^ During the Zika virus epidemic in Rio de Janeiro, Brazil, during the period 2015–2016, it was shown that neuropathies increased.^4^ However, until now there has not been a baseline of GBS cases prior to the Zika virus epidemic to provide a comparison with GBS rates reported during the epidemic. To assemble such a baseline, we investigated the extent to which the increase in GBS cases in 2015–2016 was greater or less than other increases over the two previous decades.

We compared a historical time series of inflammatory polyneuropathies, which included GBS, to time series of influenza, dengue virus, and Zika virus in the state of Rio de Janeiro, Brazil. Further, we analyzed medical charts of arbovirus infections that resulted in hospitalization with neurological disease. Our objectives were to explore whether epidemics of emerging viruses are associated with neurological hospitalizations in Brazil and whether models could be used to predict neurological sequelae from viral infections.

## 2 MATERIALS AND METHODS

**Figure 1:**
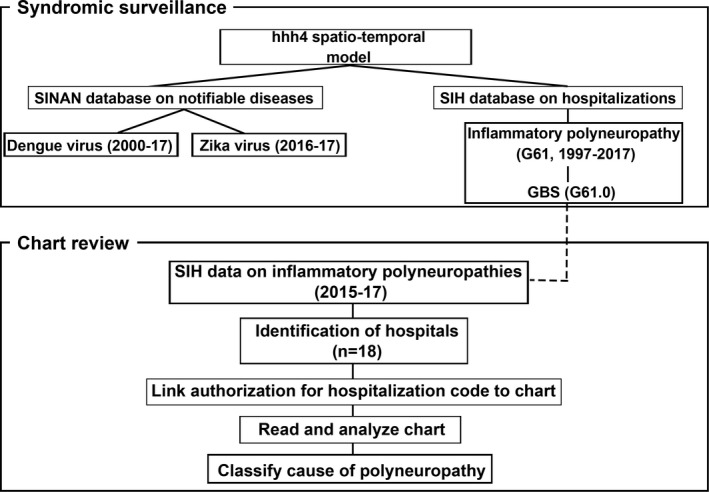
Stages of the analysis. We tallied GBS cases by analyzing a hospitalization database for 1997–2017 in the state of Rio de Janeiro (see Fig. 2). We also reviewed charts from 2015–17 in the Metro Area II to identify cases of GBS associated with arboviruses. Abbreviations: SIH, Portuguese acronym for hospitalization database; SINAN, Portuguese acronym for notifiable diseases database; G61, ICD‐10 code for inflammatory polyneuropathy; GBS, Guillain‐Barré syndrome.

**Figure 2:**
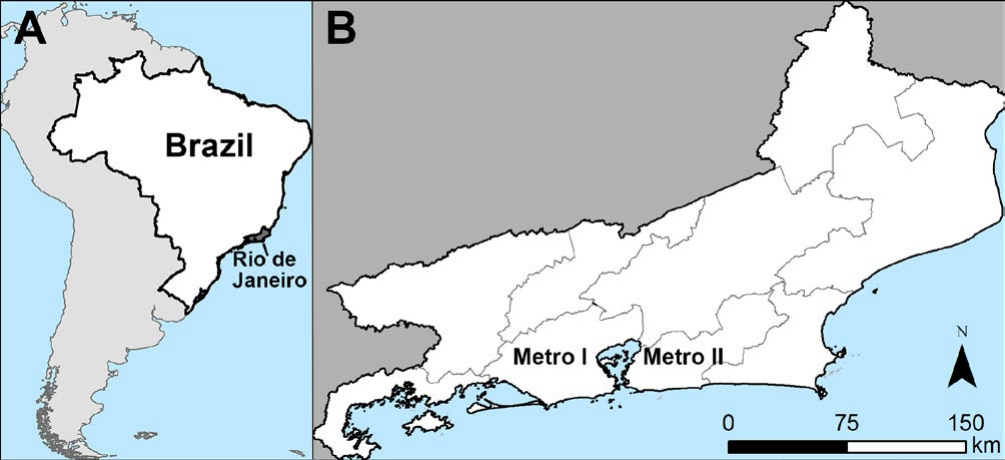
Spatial scale of analysis of syndromic surveillance data on inflammatory polyneuropathies from 1997–2017. (A) Location of Rio de Janeiro in Brazil; (B) State of Rio de Janeiro showing the nine health districts. [Colour figure can be viewed at http://www.wileyonlinelibrary.com]

We carried out an ecological study in which we observed time trends in the number of hospitalizations with inflammatory polyneuropathy (ICD‐10: G61) and Guillain‐Barré syndrome (G61.1) in the state of Rio de Janeiro reported to Brazil's hospitalization database (Sistema de Informações Hospitalares [SIH], in Portuguese) from 1997 to 2017 (Figs 1 and 2). We analyzed data on dengue virus and Zika virus from Brazil's notifiable diseases database (Sistema de Agravos de Notificação [SINAN], in Portuguese). Zika virus cases are only available in SINAN beginning in 2016 when the virus became notifiable in Brazil.

To quantify trends in GBS from 1997 to 2017, we compared the number of cases reported to SIH in a given month to the number the following month. We assessed whether the difference in the number of cases from 1 month to the next was significant using Farrington's outbreak rate test, which is a regression model that predicts the expected number of cases in a given month based on the history of cases in previous months.^5^ Next, we evaluated the extent to which cases of GBS could be explained by cases of Zika virus and Zika + dengue virus. We used the hhh4 model for areal time series of counts in the statistical software R version 3.5.1.^6^ hh4 is a multivariate model in which the number of cases of a disease in a geographic region depends on the number of cases in the region in previous months and a neighborhood matrix that represents the probability of disease spread between regions. To prepare the data for the model, we tabulated the number of cases of GBS, Zika virus, and dengue virus per month in Rio de Janeiro's nine health districts. The number of cases of GBS in district *i* in month *t* depended on the population of *i*, cases of dengue virus and Zika virus in month *t*, the number of GBS cases in district *i* in month *t*‐1, and the neighborhood matrix (supporting information Tables S1 and S2). The model included all months of the year and had sine‐cosine effects of time to reflect seasonal variation in GBS incidence. Since Zika virus and dengue virus have similar clinical presentations such as febrile illness and rash,^7^ we pooled the Zika + dengue virus cases into the category *arbovirus* cases. We also analyzed clinical cases of Zika virus separately from the Zika + dengue virus cases.

While the analysis of syndromic surveillance data was an ecological study in which we observed temporal trends in GBS, we also carried out a cross‐sectional study to assess the cause of GBS on clinical grounds. The cross‐sectional study took place in Metropolitan Area II of Rio de Janeiro because this area had the highest incidence of Zika virus in the state in 2016 (Fig. 2, supporting information Fig. S1). The study was approved by the Research Ethics Committee of the Sergio Arouca National School of Public Health, Oswaldo Cruz Foundation, CAAE (approval no. 6651851690005240), and the Pan‐American Health Organization Ethics Review Committee (approval no. PAHO–2017‐03‐0037). All participants provided written informed consent before the study began.

Metro II has a population of 1.9 million and encompasses seven municipalities on the east side of the greater Rio de Janeiro metropolitan area. From 2015 to 2017, 18 hospitals in Metro II reported inflammatory polyneuropathies to SIH. We requested authorization from the hospitals’ research departments to review charts and received permission at eight hospitals that represented 81% of admissions for inflammatory polyneuropathies. We visited these hospitals and used the authorization number from SIH to identify each patient's chart (the same patient had more than one authorization number if hospitalized repeatedly). We transcribed the physicians’ notes about clinical signs and symptoms reported by the patient. Based on this information, we used the classification system proposed by Martyn and Hughes^8^ to categorize the inflammatory polyneuropathy as either diabetes, genetic disorder, infectious disease, vaccine‐related, alcohol, or other etiology. We also made note of whether the patient had a lab test for chikungunya, dengue, or Zika virus.

## 3 RESULTS

**Figure 3:**
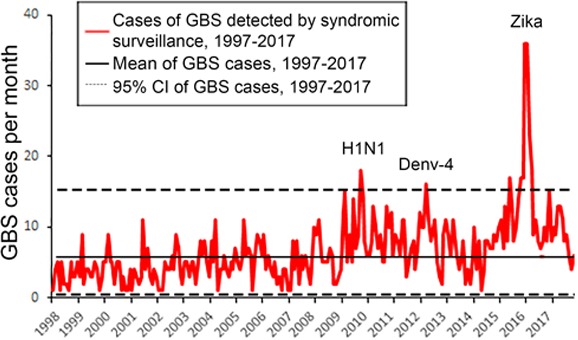
Time series of cases of GBS detected by syndromic surveillance from 1997–2017 according to the SIH database. We used the Farrington test to identify months in which the number of GBS cases increased significantly over the previous year(s). [Colour figure can be viewed at http://www.wileyonlinelibrary.com]

From 1997 to 2017, 1593 cases of GBS were reported in the state of Rio de Janeiro (annual average, 60 cases). Analysis of monthly GBS cases determined that there were significant increases in GBS from 1 month to the next for 3 years (Farrington test *P*<0.001): in 2009 (n=115 cases) contemporaneous with the introduction of H1N1^9^; in 2013 (n=85), which coincided with an epidemic of dengue virus type 4^10^; and in 2015 with the arrival of Zika virus (n=326) (Fig. 3).

There was seasonal variation in GBS incidence with 15% fewer cases during winter. Zika virus cases were a significant variable for explaining cases of GBS (hhh4 model, *t*=2.23, *P*=0.04; supporting information Table S3). The data were compatible with the null hypothesis that Zika virus cases provided a good fit to the GBS cases (calibration test *z*=0.836, *n*=189, *P*=0.403). As noted above, due to the difficulty of distinguishing Zika virus from dengue virus, we also pooled the data into the category *arboviruses*. The pooled data were not significant for explaining GBS cases (hhh4 model, *t*=1.59, *P*>0.05; supporting information Table S4). When we compared the goodness of fit of the Zika virus and Zika + dengue virus models, the former had lower errors (mean difference=0.044, permutation test *P*=0.0049).

**Figure 4:**
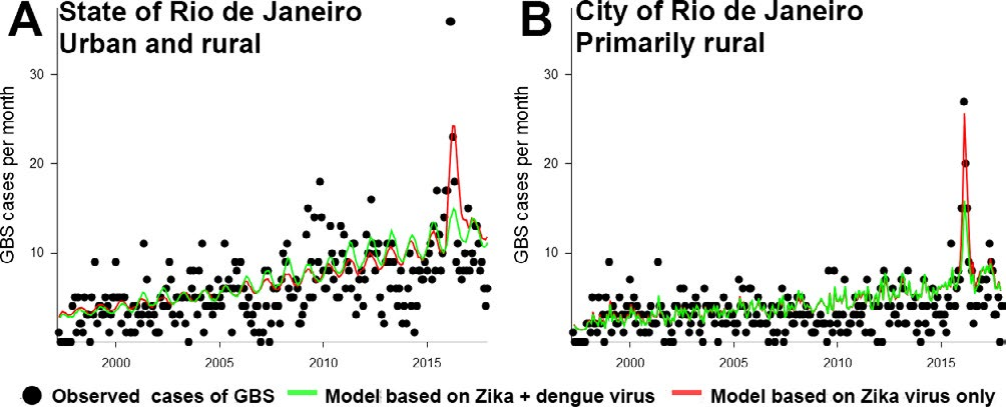
Zika cases predict cases of GBS. (A) Rio de Janeiro state; (B) Metropolitan Areas I and II. The goodness of fit of the model based on Zika + dengue virus is lower than that of the Zika virus only model. For both models, accuracy is higher in urban areas than at the scale of the state of Rio de Janeiro, which is 16% rural. [Colour figure can be viewed at http://www.wileyonlinelibrary.com]

The greatest increase in GBS from 1997 to 2017 coincided with the introduction of Zika virus in 2015–2016 (Fig. 3). A predictive model based on Zika virus cases explains the temporal pattern of GBS cases better than a model based on Zika + dengue virus (Fig. 4). An example is the metropolitan area of the city of Rio de Janeiro (Fig. 4B). The model based on Zika virus predicts a major increase in GBS cases, while the model based on Zika + dengue virus predicts a smaller increase. Dengue virus did not improve the predictive accuracy of the model because there were more than 200 000 dengue virus cases from 2001 to 2010 (supporting information Fig. S2), but few GBS cases were reported during this period. The fit of the Zika virus model to the GBS cases is poorer for the entire state of Rio de Janeiro (Fig. 4A) than for the metropolitan area of the city of Rio de Janeiro (Fig. 4B).

We reviewed charts at eight hospitals, which encompassed 80 (82.3%) of the 97 admissions for inflammatory polyneuropathies. These 80 admissions corresponded to 50 patient charts because some patients were admitted repeatedly. The most frequent causes of inflammatory polyneuropathy were arboviruses (n=14, 28.0%), followed by other viruses (18.0%) (Table 1). Table 2 reports antecedent conditions and symptoms of inflammatory neuropathy associated with arboviruses. The average age of the patients was 42 years (interquartile range, 27–51 years) and the male:female ratio was 0.67. Of the 14 patients with inflammatory polyneuropathies caused by arboviruses, 12 had classic GBS (85.7%), one had the Miller‐Fisher variant of GBS, and another had progressive idiopathic neuropathy instead of GBS or an arboviral infection.

**Table 1:** Causes of inflammatory polyneuropathies in individuals (n=50) hospitalized in Metropolitan Area II from 2015–2017 based on the categories of Martyn and Hughes^8^ plus arboviruses

Causes of inflammatory neuropathy	No. (%)
I. Diabetic neuropathy	5 (10.0)
II. Hereditary neuropathy	
Familial amyloid polyneuropathy	1 (2.0)
Demyelinating motor sensory polyneuropathy	2 (4.0)
III. Infectious and inflammatory neuropathy	
Demyelinating chronic inflammatory polyneuropathy	3 (6.0)
Hepatitis	1 (2.0)
Arbovirus	14 (28.0)
HIV	2 (4.0)
Other viruses	9 (18.0)
Respiratory infection	1 (2.0)
IV. Alcoholism	1 (2.0)
V. Vaccination	3 (6.0)
VI. Other	
Unknown	8 (16.0)

**Table 2:** Signs and symptoms of inflammatory polyneuropathies associated with arboviruses (n=14)

Signs and symptoms of neuropathy	No. (%)
Musculoskeletal symptoms	
Arthralgia/myalgia	5 (35.7)
Back pain	2 (14.3)
Nervous system symptoms	
Paresthesia	7 (50.0)
Headache	2 (14.3)
Paraparesis	7 (50.0)
Paraplegia	2 (14.3)
Tetraparesis	5 (35.7)
Tetraplegia	2 (14.3)
Facial paralysis	5 (35.7)
Weakness	9 (64.3)
Dysarthria	1 (7.1)
Dysphagia	2 (14.3)
Gait disturbance	2 (14.3)
Encephalitis	1 (7.1)
Respiratory symptoms	
Shortness of breath	1 (7.1)
Gastrointestinal symptoms	
Incontinence	2 (14.3)

Of the 14 cases of inflammatory polyneuropathy associated with arboviruses, nine were treated with immunoglobulin. Of the 14 cases with inflammatory polyneuropathy and arboviruses, Zika virus infection was confirmed by reverse transcriptase polymerase chain reaction in one (6.7%) patient; one patient had chikungunya virus confirmed by serology and one patient was positive for dengue virus by laboratory exams. The other 11 (78.6%) patients were diagnosed with arbovirus infection based on clinical symptoms. Of the 14 patients, 8 (57.1%) presented with cutaneous rash, 7 (50.0%) with fever, and 3 (21.4%) conjunctivitis (Table 2). A total of 9 (64.3%) of these 14 patients were treated with intravenous immunoglobulin. No patient was treated with plasma exchange. Five (55.6%) of the nine treated with intravenous immunoglobulin were transferred to other hospitals because the treatment was not available at the first hospital to which they were admitted. As of 2018, 12 (85.7%) of the 14 had no motor deficits, one still had facial paralysis, and one remained quadriplegic.

## 4 DISCUSSION

While it is well established that infection with pathogens that cause inflammation can result in neuropathy,^8^ there have been few epidemiological studies of neuropathies in Brazil. Our results indicated that increases in GBS were related to the epidemics of H1N1 in 2009, dengue virus type 4 in 2013, and Zika virus in 2015. In 2009, a novel reassortant influenza A subtype H1N1 spilled over from swine to immunologically naïve human populations.^11^ In the state of Rio de Janeiro, H1N1's arrival was followed by an increase in GBS. The increases in GBS in 2013 and 2015 coincided with the introduction of novel arboviruses. Although the H1N1 epidemic in 2009 coincided with an increase in GBS, none of the patients whose charts we reviewed in 2015–2017 had symptoms of influenza or recent influenza vaccination. Epidemiological studies have not demonstrated a relationship between influenza vaccination and increased risk of GBS.^12^ GBS incidence may have been lower in the winter because arboviral infections happen predominantly in the summer, spring, and fall.

These results support previous findings indicating an association between arboviruses and neuropathies in Brazil during the Zika virus epidemic.^4^, ^13^, ^14^, ^15^ The rates of rash, facial paralysis, and paraparesis in patients with GBS in Metropolitan Area II are similar to those previously reported for GBS cases in Latin America and the Caribbean.^3^, ^4^, ^15^, ^16^, ^17^ Our classification of the cause of inflammatory polyneuropathies was based on signs and symptoms reported by the patient and investigated by physicians. However, the causes are not mutually exclusive. For example, a patient could be diabetic, HIV seropositive, use corticosteroids, have alcoholism, and have a silent Zika virus infection, as up to 60% of cases are asymptomatic.^18^ Although the most frequent causes of GBS around the world are *Campylobacter*,* Cytomegalovirus*, and influenza, in our chart review no patient was diagnosed with them. However, for nine patients the diagnosis was “other viruses.” The other viruses may have represented cases of *Campylobacter*,* Cytomegalovirus*, or influenza.

The Zika virus epidemic in Brazil is unique in that Brazil is the only country with simultaneous transmission of four medically important arboviruses: chikungunya, dengue virus, Zika virus, and yellow fever virus. All four can cause neurological and neuromuscular complications including GBS.^19^, ^20^ Distinguishing between chikungunya, dengue virus, and Zika virus is difficult because of similar signs and symptoms and the low sensitivity and specificity of laboratory assays.^21^

Due to the difficulty of distinguishing among arboviruses in the analysis of syndromic surveillance, we pooled suspected cases of Zika virus and dengue virus into the category of arboviruses. Nevertheless, the model based on Zika alone explained GBS cases better than the model based on Zika + dengue virus (arboviruses). Adding dengue virus to the model failed to improve goodness of fit because the epidemics that occurred in the early 2000s were not accompanied by an increase in GBS, and the incidence of dengue virus was low in 2015–2016.^22^ The predictive model was more accurate in the metropolitan area of the city of Rio de Janeiro, which is primarily urban, than across the whole state of Rio de Janeiro, which encompasses urban and rural areas. Poorer accuracy in rural areas could be due to less intensive epidemiological surveillance or fewer *Aedes* mosquitos in these areas.

Chart review indicated that suspected Zika virus cases were seldom confirmed by laboratory exams. To this extent, we can only report that patients had some type of arbovirus, but cannot determine whether it was chikungunya, dengue, or Zika virus. Differential diagnosis of *Flavivirus* species is complicated because commercial tests for dengue virus cross‐react to Zika virus antibodies and vice versa.^23^, ^24^

The present study has several implications for planning the treatment of neurological complications of arboviruses. Five (55.6%) of the nine patients treated with intravenous immunoglobulin were transferred to a different hospital. On the other hand, none of the patients admitted to a neurology reference hospital were transferred. It appears that only reference hospitals had access to intravenous immunoglobulin, whereas the treatment was not available in other public hospitals. To fill this gap, there is a need for public health decision makers to assure the supply of intravenous immunoglobulin in the public health institutions network. This would likely reduce the need for transfers, which worsen GBS patient outcomes and are costly.^25^ We found that it is possible to predict the timing and location of hospitalizations with GBS with a statistical model using data on arbovirus cases. GBS typically presents 1–2 weeks after infection.^1^ Using our models, if syndromic surveillance detects an increase in arboviruses in a health district, decision makers would have 1–2 weeks to allocate intravenous immunoglobulin to the district's hospitals to prepare for GBS cases.

A limitation of this study is the diagnosis of suspected arbovirus infections. Diagnosis was primarily based on clinical signs and symptoms, which could not be confirmed by laboratory diagnosis in 78.6% of cases. Further, as this study was hospital based, we would not have detected inflammatory polyneuropathies that did not result in hospitalization. In addition, since the analysis was limited to the state of Rio de Janeiro, which had a high incidence of Zika virus, it is unknown whether our conclusions apply to other regions where outbreaks were smaller.

Predictive modeling is useful for forecasting future observations, explaining processes, and testing theories.^26^ We utilized predictive modeling to test the hypothesis that an emerging virus, namely Zika virus, could explain cases of GBS in the population of Rio de Janeiro. The model accurately predicted the pattern of occurrences of GBS across the state. Further, the model consisting of Zika virus alone outperformed the model with all arboviruses, which underscores the important role that this emerging virus plays in the occurrence of GBS.

## AUTHOR CONTRIBUTIONS

JRA and TLF contributed to study design, data analysis, and manuscript writing. BBSL, OCP, and PCS contributed to study design. HLFP and RDM performed field work and chart review. JMCF and CCBP contributed significantly to the patient database and chart review. KNS contributed to manuscript writing. OJMN: contributed significantly to the patient database and manuscript writing.

## CONFLICTS OF INTEREST

The authors have no conflicts of interest.

## Supporting information


**Figure S1.** Zika virus incidence in the state of Rio de Janeiro by health district.
**Figure S2.** Clinical cases of dengue virus in the state of Rio de Janeiro, 2001–2017 according to SINAN.
**Table S1.** Notation for the hhh4 time series model to predict the number of cases of GBS per health district in each month.
**Table S2.** Formulation of the hhh4 model. α^ν^, β^ν^, γ, δ, α^λ^, β^λ^, ϕ_it_ e ψ_i_ were estimated from the data using the library SURVEILLANCE in R.^6^
**Table S3.** hhh4 model based on Zika virus cases in the state of Rio de Janeiro, 1997–2017. Values in bold indicate *P*<0.05.
**Table S4.** hhh4 model based on Zika + dengue virus in the state of Rio de Janeiro, 1997–2017. Values in bold indicate *P*<0.05.

## References

[1] Willison HJ , Jacobs BC , van Doorn PA . Guillain‐Barre syndrome. Lancet. 2016;388:717–727. 26948435 10.1016/S0140-6736(16)00339-1

[2] van den Berg B , Walgaard C , Drenthen J , Fokke C , Jacobs BC , Van Doorn PA . Guillain‐Barre syndrome: Pathogenesis, diagnosis, treatment and prognosis. Nat Rev Neurol. 2014;10:469–482. 25023340 10.1038/nrneurol.2014.121

[3] Rozé B , Najioullah F , Fergé J‐L , et al. Guillain‐Barré syndrome associated with Zika virus infection in martinique in 2016: A prospective study. Clin Infect Dis. 2017;65:1462–1468. 29020245 10.1093/cid/cix588

[4] Mehta R , Soares CN , Medialdea‐Carrera R , et al. The spectrum of neurological disease associated with Zika and chikungunya viruses in adults in Rio de Janeiro, Brazil: A case series. PLoS Negl Trop Dis. 2018;12:e0006212. 29432457 10.1371/journal.pntd.0006212 PMC5837186

[5] Farrington CP , Andrews NJ , Beale AD , Catchpole MA . A statistical algorithm for the early detection of outbreaks of infectious disease. J Royal Stat Soc Ser A. 1996;159:547–563.

[6] Meyer S , Held L , Hohle M . Spatio‐temporal analysis of epidemic phenomena using the R package surveillance. J Stat Softw. 2017;77:1–55.

[7] Braga JU , Bressan C , Dalvi APR , et al. Accuracy of Zika virus disease case definition during simultaneous Dengue and Chikungunya epidemics. PLoS ONE. 2017;12:e0179725. 28650987 10.1371/journal.pone.0179725 PMC5484469

[8] Martyn CN , Hughes RA . Epidemiology of peripheral neuropathy. J Neurol Neurosurg Psychiatry. 1997;62:310–318. 9120441 10.1136/jnnp.62.4.310 PMC1074084

[9] Souza TML , Salluh JIF , Bozza FA , et al. H1N1pdm influenza infection in hospitalized cancer patients: Clinical evolution and viral analysis. PLoS ONE. 2010;5:e14158. 21152402 10.1371/journal.pone.0014158 PMC2994772

[10] Heringer M , Souza TMA , Lima MDRQ , et al. Dengue type 4 in Rio de Janeiro, Brazil: Case characterization following its introduction in an endemic region. BMC Infect Dis. 2017;17:410. 28599640 10.1186/s12879-017-2488-4 PMC5466795

[11] Novel Swine‐Origin Influenza A (H1N1) Virus Investigation Team , Dawood FS , Jain S , et al. Emergence of a novel swine‐origin influenza A (H1N1) virus in humans. New Engl J Med. 2009;360:2605–2615. 19423869 10.1056/NEJMoa0903810

[12] Wachira VK , Peixoto HM , de Oliveira MRF . Systematic review of factors associated with the development of Guillain‐Barre syndrome 2007‐2017: What has changed? Trop Med Int Health. 2019;24:132–142. 30444562 10.1111/tmi.13181

[13] Keesen TSL , de Almeida RP , Gois BM , et al. Guillain‐Barre syndrome and arboviral infection in Brazil. Lancet Infect Dis. 2017;17:693–694. 28653634 10.1016/S1473-3099(17)30333-X

[14] Malta JM , Vargas A , Leite PL , et al. Guillain‐Barre syndrome and other neurological manifestations possibly related to Zika virus infection in municipalities from Bahia, Brazil, 2015. Epidemiol Serv Saude. 2017;26:9–18. 28226004 10.5123/S1679-49742017000100002

[15] Styczynski AR , Malta JMAS , Krow‐Lucal ER , et al. Increased rates of Guillain‐Barré syndrome associated with Zika virus outbreak in the Salvador metropolitan area, Brazil. PLoS Negl Trop Dis. 2017;11:e0005869. 28854206 10.1371/journal.pntd.0005869 PMC5595339

[16] Arias A , Torres‐Tobar L , Hernandez G , et al. Guillain‐Barre syndrome in patients with a recent history of Zika in Cucuta, Colombia: A descriptive case series of 19 patients from December 2015 to March 2016. J Crit Care. 2017;37:19–23. 27610587 10.1016/j.jcrc.2016.08.016

[17] Salinas JL , Major CG , Pastula DM , et al. Incidence and clinical characteristics of Guillain‐Barre syndrome before the introduction of Zika virus in Puerto Rico. J Neurol Sci. 2017;377:102–106. 28477675 10.1016/j.jns.2017.04.006

[18] Haby MM , Pinart M , Elias V , Reveiz L . Prevalence of asymptomatic Zika virus infection: A systematic review. Bull World Health Organ. 2018;96:402–413D. 29904223 10.2471/BLT.17.201541 PMC5996208

[19] Carod‐Artal F , Wichmann O , Farrar J , Gascon J . Neurological complications of dengue virus infection. Lancet Neurol. 2013;12:906–919. 23948177 10.1016/S1474-4422(13)70150-9

[20] Chandak NH , Kashyap RS , Kabra D , et al. Neurological complications of Chikungunya virus infection. Neurol India. 2009;57:177–180. 19439849 10.4103/0028-3886.51289

[21] Musso D , Gubler DJ . Zika virus. Clin Microbiol Rev. 2016;29:487–524. 27029595 10.1128/CMR.00072-15 PMC4861986

[22] Fuller TL , Calvet G , Genaro Estevam C , et al. Behavioral, climatic, and environmental risk factors for Zika and Chikungunya virus infections in Rio de Janeiro, Brazil, 2015‐16. PLoS ONE. 2017;12:e0188002. 29145452 10.1371/journal.pone.0188002 PMC5690671

[23] Kikuti M , Tauro LB , Moreira PSS , et al. Diagnostic performance of commercial IgM and IgG enzyme‐linked immunoassays (ELISAs) for diagnosis of Zika virus infection. Virol J. 2018;15:108. 30005683 10.1186/s12985-018-1015-6 PMC6045861

[24] Felix AC , Souza NCS , Figueiredo WM , et al. Cross reactivity of commercial anti‐dengue immunoassays in patients with acute Zika virus infection. J Med Virol. 2017;89:1477–1479. 28229481 10.1002/jmv.24789

[25] van Leeuwen N , Lingsma HF , Vanrolleghem AM , et al. Hospital admissions, transfers and costs of Guillain‐Barre syndrome. PLoS ONE. 2016;11:e0143837. 26859880 10.1371/journal.pone.0143837 PMC4747559

[26] Shmueli G . To explain or to predict? Stat Sci. 2010;25:289–310.

