# Supplementary material for: Neurological complications associated with emerging viruses in Brazil
Source: Int J Gynaecol Obstet. 2020 Jan 23;148(Suppl 2):70–5. doi: 10.1002/ijgo.13050 (PMC7065065; doi:10.1002/ijgo.13050)

**Supporting information**

**Supporting information S1.** Notation for the hhh4 time series model to predict the number of cases of GBS per health district in each month.

| Sets | |
| --- | --- |
| $i$ | Health districts of the state of Rio de Janeiro (n=9) |
| $t$ | Months, April 1997 to December 2017 |
| Parameters | |
| $Y_{it}$ | Number of cases of GBS in district *i* in month *t*. *Y_it_* is a random variable with a negative binomial distribution. |
| $\mu_{it}$ | Mean of the distribution of *Y_it_* |
| ${}_{i}$ | Overdispersion of the distribution of *Y_it_* |
| $e_{i}$ | Population of district *i* |
| $v_{t}$ | Effect of seasonal variation on the number of GBS cases in month *t* |
| ${}_{it}$ | Coefficient representing the effect of arbovirus cases in district *i* in month *t* |
| $x_{it}$ | Number of arbovirus cases in district *i* in month *t* |
| ${}_{it}$ | Movement of GBS cases from district *i* to neighboring districts in month *t* |
| $w_{ij}$ | Transmission matrix from district *i* to district *j*. We assume district *i* can receive cases from neighboring districts. |
| ω | Period of the seasonality parameter. We used ω=12 because the syndromic surveillance data were reported on a monthly basis. |

**Supporting information S2.** Formulation of the hhh4 model. $\alpha$, $\beta$, $\gamma$, $\delta$, $\alpha$, $\beta$, ${}_{it}$ e ${}_{i}$were estimated from the data using the library SURVEILLANCE in R [6].

| $\mu_{it}$ | ${=e}_{i}v_{t}$ +${}_{it}Y_{i,t-1}$+${}_{it}\sum_{j\neq i} w_{ji}Y_{i,t-1}$ | (1) |
| --- | --- | --- |
| $\log\left( v_{t} \right)$ | ${=\alpha}+\beta t+\gamma\sin\left( \omega t \right)+\delta\cos(\omega t)$ | (2) |
| $\log\left( {}_{it} \right)$ | ${=\alpha}+\beta\log(x_{it})$ | (3) |

**Supporting information S3.** Zika virus incidence in the state of Rio de Janeiro by health district. Bars represent monthly incidence in 2016. The highest incidence of Zika virus was in Metropolitan Area II. We calculated incidence using Zika virus cases confirmed by reverse transcriptase PCR according to SINAN: Source [22] and <http://portalsinan.saude.gov.br/o-sinan> (Accessed December 17, 2018). Samples were screened from January to April 2016. The panels represent the health districts of Rio de Janeiro. MI = Metro I; MII = Metro II; NO = Noroeste; N = Norte; MP = Médio Paraíba; BL = Baixada Litorânea; S = Serrana; BIG = Baía da Ilha Grande; CS = Centro-Sul.


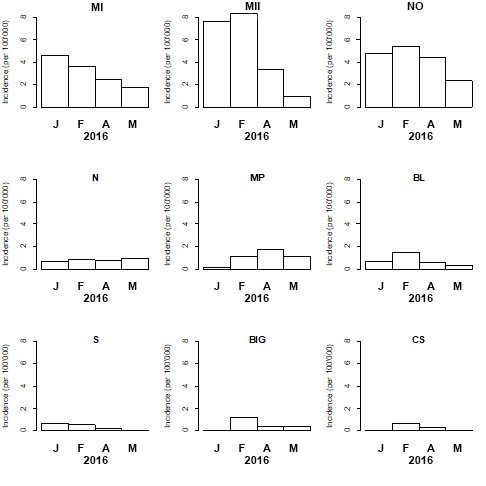


**Supporting information S4.** hhh4 model based on Zika virus cases in the state of Rio de Janeiro, 1997–2017. Values in bold indicate *P*<0.05.

| Parameter | Definition | Estimate | Standard error | *t* | *df* | *P* value |
| --- | --- | --- | --- | --- | --- | --- |
| $\alpha$ | Intercept of λit | –1.162 | 0.0849 | -13.687 | 8 | **<0.001** |
| $\beta$ | Effect of Zika virus on GBS cases | 0.0974 | 0.0437 | 2.227 | 8 | **0.04** |
| ${}_{it}$ | Effect of movement from neighboring districts on GBS cases | –3.599 | 0.214 | -16.841 | 8 | **<0.001** |
| $\alpha$ | Intercept of vt | –15.922 | 0.110 | -144.221 | 8 | **<0.001** |
| $\beta$ | Effect of seasonality on the number of GBS cases | 5.53×${10}^{-3}$ | 7.16×${10}^{-4}$ | 7.724 | 8 | **<0.001** |
| $\gamma$ | Effect of seasonality on the number of GBS cases | –0.0316 | 0.071 | -0.445 | 8 | 0.346 |
| $\delta$ | Effect of seasonality on the number of GBS cases | 0.1294 | 0.071 | 1.822 | 8 | 0.0812 |
| ${}_{i}$ | Overdispersion | 0.444 | 0.0628 | 7.07 | 8 | **<0.001** |

**Supporting information S5.** hhh4 model based on Zika+dengue virus in the state of Rio de Janeiro, 1997–2017. Values in bold indicate *P*<0.05.

| Parameter | Definition | Estimate | Standard error | *t* | *df* | *P* value |
| --- | --- | --- | --- | --- | --- | --- |
| $\alpha$ | Intercept of λit | –1.31968 | 0.1509 | –8.74539 | 8 | **<0.001** |
| $\beta$ | Effect of Zika virus on GBS cases | 0.0481 | 0.0303 | 1.587459 | 8 | 0.113 |
| ${}_{it}$ | Effect of movement from neighboring districts on GBS cases | –3.5698 | 0.21 | –16.999 | 8 | **<0.001** |
| $\alpha$ | Intercept of vt | –15.931 | 0.1107 | –143.911 | 8 | **<0.001** |
| $\beta$ | Effect of seasonality on the number of GBS cases | 0.005609 | 0.000717 | 7.822873 | 8 | **<0.001** |
| $\gamma$ | Effect of seasonality on the number of GBS cases | –0.0413 | 0.07108 | –0.58104 | 8 | 0.321 |
| $\delta$ | Effect of seasonality on the number of GBS cases | 0.12699 | 0.072097 | 1.761377 | 8 | 0.0885 |
| ${}_{i}$ | Overdispersion | 0.4547 | 0.06298 | 7.219752 | 8 | **<0.001** |

**Supporting information S6.** Clinical cases of dengue virus in the state of Rio de Janeiro, 2001–2017 according to SINAN.


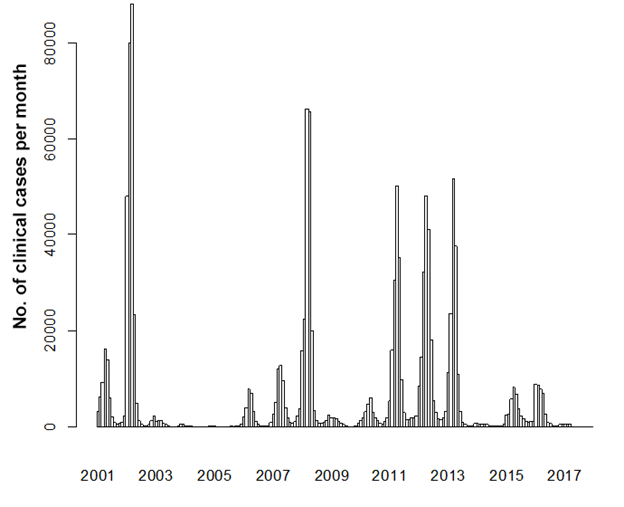

Supplement: Supplementary file 1 — Figure S1. Zika virus incidence in the state of Rio de Janeiro by health district. Figure S2. Clinical cases of dengue virus in the state of Rio de Janeiro, 2001–2017 according to SINAN. Table S1. Notation for the hhh4 time series model to predict the number of cases of GBS per health district in each month. Table S2. Formulation of the hhh4 model. αν, βν, γ, δ, αλ, βλ, ϕit e ψi were estimated from the data using the library SURVEILLANCE in R.6 Table S3. hhh4 model based on Zika virus cases in the state of Rio de Janeiro, 1997–2017. Values in bold indicate P<0.05. Table S4. hhh4 model based on Zika + dengue virus in the state of Rio de Janeiro, 1997–2017. Values in bold indicate P<0.05. [file IJGO-148-70-s001.docx]
